# Pancreatic acinar differentiation is guided by differential laminin deposition

**DOI:** 10.1038/s41598-019-39077-6

**Published:** 2019-02-25

**Authors:** Charlotte Heymans, Jonathan Degosserie, Catherine Spourquet, Christophe E. Pierreux

**Affiliations:** Cell Biology Unit, de Duve Institute, UCLouvain, Woluwe, Belgium

## Abstract

Endothelial cells play multiple roles during pancreas organogenesis. First, they are required to instruct endoderm-derived pancreatic progenitor cells to initiate branching morphogenesis. Later, blood vessels promote β-cell differentiation but also limit acinar development. In this work, we show how endothelial cells might signal to pancreatic progenitors and spatially regulate acinar differentiation. Using an *ex vivo* culture system of undifferentiated E12.5 pancreata, we demonstrate that embryonic endothelial progenitor cells and their conditioned medium prevent the expression of two members of the pro-acinar transcriptional PTF1L-complex. This effect is not mediated by SPARC, a protein abundantly released in the medium conditioned by endothelial progenitors. On the contrary, heterotrimeric laminin-α1β1γ1, also produced by endothelial progenitor cells, can repress acinar differentiation when used on its own on pancreatic explants. Lastly, we found that laminin-α1 is predominantly found *in vivo* around the pancreatic trunk cells, as compared to the tip cells, at E14.5. In conclusion, we propose that expression or deposition of laminin-α1β1γ1 around the trunk cells, where blood vessels are predominantly localized, prevent acinar differentiation of these cells. On the contrary, transient decreased expression or deposition of laminin-α1β1γ1 around the tip cells would allow PTF1L-complex formation and acinar differentiation.

## Introduction

The pancreas is an amphicrine gland composed of an endocrine compartment involved in the regulation of glycaemia, and an exocrine compartment implicated in digestion. Endocrine cells form the islets of Langerhans and produce hormones such as insulin and glucagon. Two types of exocrine cells can be distinguished: acinar and ductal cells. The pyramidal-shaped acinar cells are closely associated through junctional proteins to form open ovoid structures called acini. These cells produce and secrete inactive digestive zymogens, such as Amylase and Carboxypeptidase A (CPA), in the central lumen of the acini, wherefrom they are collected and transported through a network of ducts converging towards the duodenum^1^.

The pancreas develops from the endoderm through a multi-step process. The first step, called the specification, occurs around embryonic day (E) 8.5 and is characterized by the expression of the transcription factor PDX1 in some cells of the mouse foregut endoderm. The specified cells are multipotent progenitor cells (MPC) that proliferate intensively to form the ventral and dorsal pancreatic buds. These two buds will eventually fuse. Starting at E11.5, the developing pancreas expands and branches extensively. Based on the differential expression of transcription factors and the localization of MPC within the proliferating mass, two cell types can progressively be distinguished. On the one hand, SOX9^+^ trunk cells are localized in the center of the developing pancreas and will later give rise to ductal and endocrine cells. On the other hand, tip cells, expressing PTF1A and CPA, are found at the periphery of the organ^2^. The faster division rate of the tip cells, generating a trunk cell and a new peripheral tip cell, leads to the formation of branches growing in the surrounding mesenchyme. After E14.5, the tip cells progressively differentiate into exocrine acinar cells. The switch from tip to acinar cell is regulated by a change in the PTF1 trimeric transcriptional complex. In pancreatic tip cells, PTF1A binds to RBPJ and another basic helix-loop-helix protein to form the trimeric PTF1J-complex. This complex controls the expression of several genes, among which *Rbpjl*. With time, RBPJL accumulates and progressively replaces RBPJ within the PTF1J-complex, thereby forming a different PTF1L-complex. This triggers a switch in the PTF1-complex target genes that initiates acinar differentiation and digestive enzyme (e.g. Amylase) production^3,4^. Cell-autonomous factors thus guide pancreas development and acinar differentiation, but the control by extrinsic factors is less understood.

Early stages of pancreatic development have been shown to be controlled by the notochord^5^ and then by the dorsal aortas when their fusion separates the notochord from the endoderm^6^. The group of Zaret further showed that VEGFR2 null mice, lacking aortic endothelial cells, fail to express PTF1A in the endoderm and do not form the dorsal pancreatic bud^7^. Later in development, endothelial cells were also shown to control endocrine differentiation^8^. Transgenic Pdx1-driven expression of the angiogenic factor VEGF-A induced a massive recruitment of endothelial cells, followed by an increase of insulin producing cells^6^. It was further demonstrated that β-cells secrete VEGF-A and that recruited endothelial cells assemble a basement membrane composed of collagen type IV and laminins. Basement membrane is detected by β1-containing integrin heterodimers localized on β-cells, and this triggers insulin expression^9^. More recently, Magenheim *et al*. reported that pancreatic hypervascularization negatively affects pancreatic branching morphogenesis and expansion, and maintains acinar progenitors in an undifferentiated state^10^. In addition, abnormal pancreatic growth was observed in sphingosine-1-phosphate deficient mice developing hypervascularization^11^. Blood vessels thus also regulate pancreatic branching and growth. We further demonstrated that endothelial cells localization around the pancreatic epithelium regulates acinar differentiation. Indeed, during branching morphogenesis, endothelial cells are recruited close to the trunk cells, because these cells express VEGF-A, and remain at a distance from the tip cells that do not express VEGF-A. Using cultured pancreata we showed that hypovascularization promoted, while hypervascularization prevented acinar differentiation. Finally, we demonstrated that forced expression of VEGF-A in tip cells (using Ptf1a/Ela-VEGF-A mice), induced endothelial cell recruitment around tip cells and inhibition of acinar cell differentiation^12^.

In this study, we used *ex vivo* cultured pancreatic explants to better understand how endothelial cells regulate acinar differentiation. We found that endothelial cells regulate acinar differentiation in a contact-independent manner by releasing soluble factors in their environment and prevent expression of the pro-acinar PTF1L components, RBPJL and PTF1A. Our data further suggest that laminin-α1β1γ1 preferential deposition around the trunk cells, could prevent the acinar differentiation program in those pancreatic cells, but not in tip cells.

## Results

### Pancreatic explants develop and differentiate *ex vivo*

**Figure 1:**
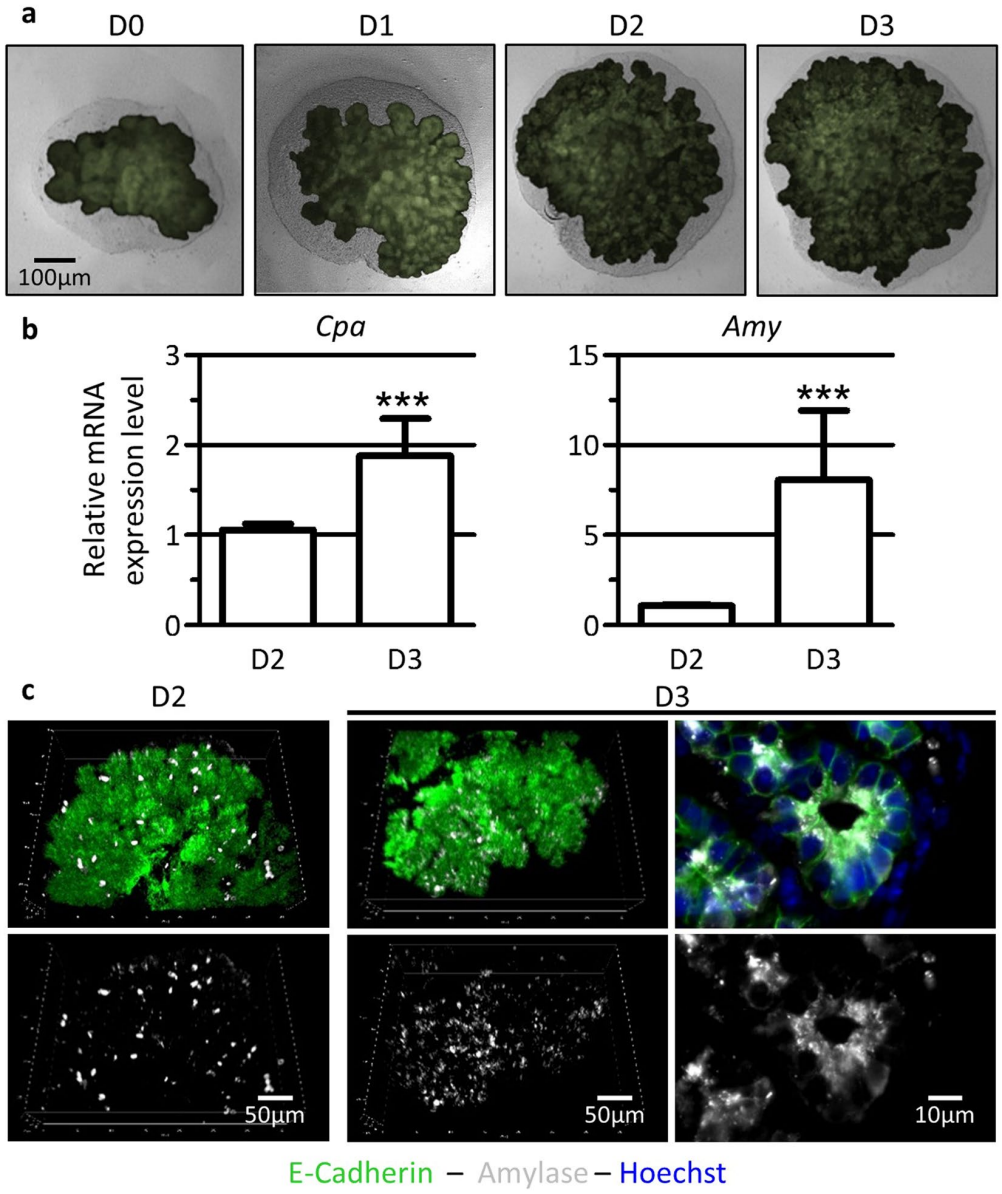
Pancreatic explants, cultured *ex vivo*, grow and differentiate. (**a**) Phase contrast and fluorescence imaging of pancreatic explants dissected at embryonic day (E)12.5 (corresponding to day (D) of culture 0), and cultured up to 3 days on microporous filters (D1-D2-D3). The pancreatic epithelium (green in Pdx1-GFP embryos) develops and progressively invades the surrounding mesenchyme (grey). (**b**) RT-qPCR analysis of acinar markers *Cpa* and *Amy* compared to *β-actin*, used as housekeeping gene. The expression of acinar markers increases from D2 to D3. (Mann-Whitney: ***p < 0.001). (**c**) Whole-mount immunofluorescence for Amylase-expressing cells (white) within the pancreatic epithelium (E-Cadherin, green). Nuclei are stained in blue (Hoechst^+^, right panel). The number of Amylase^+^ cells (white) increases from D2 to D3.

We have previously shown that endothelial cells limit acinar differentiation *in vivo* and *ex vivo*^12^. To better understand this regulation, we further used an *ex vivo* culture system of pancreatic explants that reproduce pancreatic development^13^. Pancreatic explants were micro-dissected at embryonic (E) day 12.5 and cultured on a microporous filter floating on culture medium for 2 or 3 days. The culture duration chosen corresponds to the time necessary for E12.5 pancreatic progenitors to transit from an undifferentiated to a differentiated state. We used pancreata from Pdx1-GFP transgenic embryos to visualize pancreatic epithelial growth along the culture (Fig. 1a). The epithelium (green) can thus be distinguished from the surrounding unlabeled mesenchyme (grey). At E12.5 (corresponding to culture day (D) 0) we observed a poorly branched epithelium, surrounded by mesenchyme. Along the culture (from D1 to D3), the epithelium expanded and developed branches that invaded the mesenchyme, indicating branching morphogenesis. To evaluate acinar differentiation, we analyzed the expression of the tip-and-acinar cell marker Carboxypeptidase A (*Cpa*), and of the mature acinar cell marker Amylase (*Amy*) (Fig. 1b). As acinar gene expression increases *in vivo* from E14.5 and E15.5 (Suppl. Figure S1), we compared explants cultured for 2 days (D2 = E12.5 + 2 days) with explants cultured for 3 days (D3 = E12.5 + 3 days, Fig. 1b). By RT-qPCR, we observed a ± 2-fold increase in *Cpa* expression and a ± 7-fold increase in *Amy* expression from D2 to D3. This expression profile qualitatively mimics the changes in *Cpa* and *Amy* mRNA levels observed *in vivo* from E14.5 to E15.5 (Suppl. Figure S1). Acinar differentiation was also assessed by whole-mount immunofluorescence (Fig. 1c). We used the epithelial marker E-Cadherin to visualize the pancreatic epithelium (green) and Amylase as acinar reporter (white). At D2, Amylase was found in a limited number of acinar cells, mainly located at the periphery of the explant. One day later (D3), the number of Amylase^+^ cells was substantially increased. At high magnification, we can appreciate the subcellular localization of Amylase in the apical region of the acinar cells, above the nucleus and facing the lumen. Altogether, these data indicate that this *ex vivo* culture system allows pancreatic growth, branching and acinar differentiation over the culture time, i.e. from E12.5 (D0) to “E15.5” (D3).

### Co-culture of endothelial progenitor cells with pancreatic explants limits acinar differentiation

**Figure 2:**
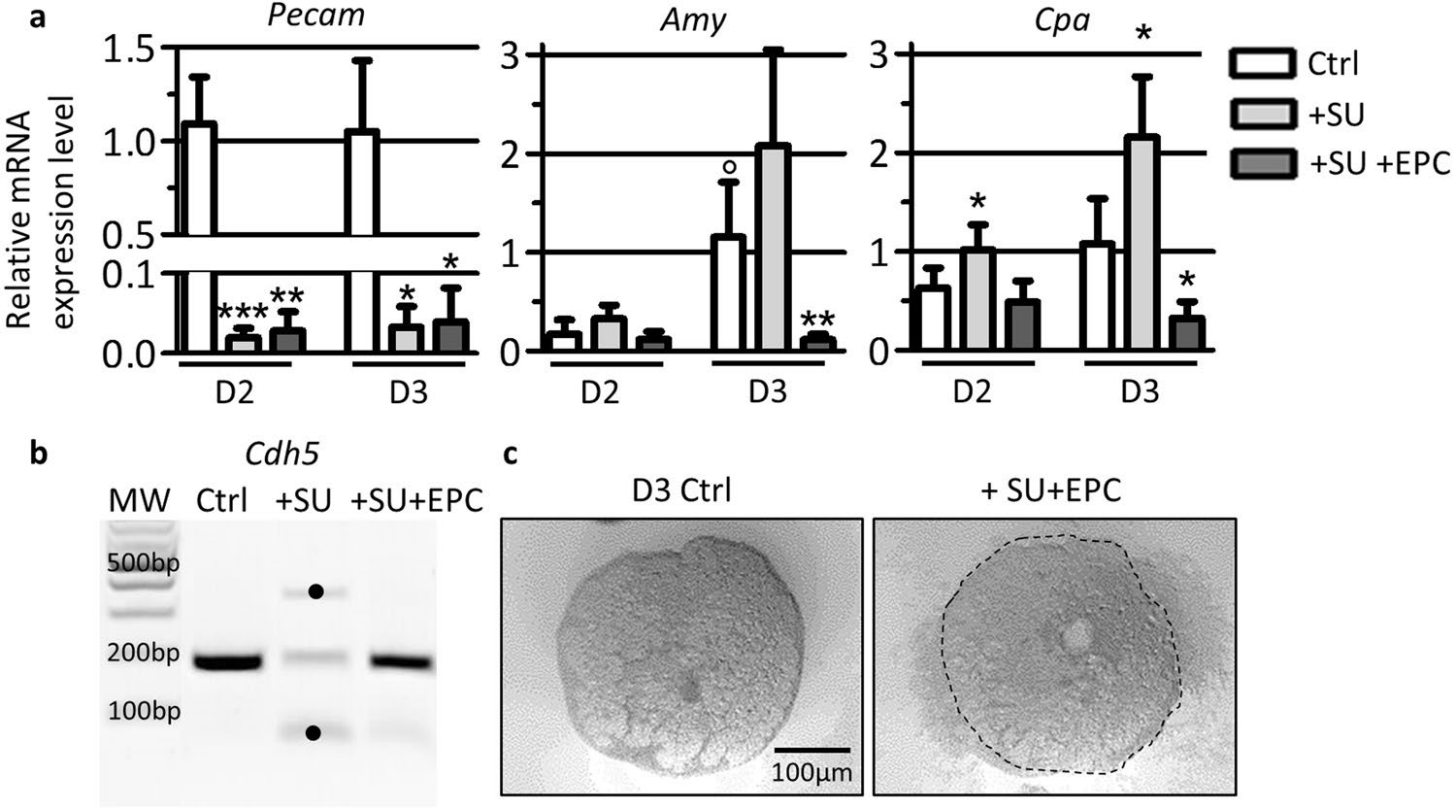
Endothelial cell ablation stimulates, while addition represses expression of acinar genes. (**a**) RT-qPCR analysis of endothelial markers *Pecam* and acinar markers *Cpa* and *Amy* reported to *β-actin* in explants cultured for 2 and 3 days in the presence of control medium (Ctrl), supplemented with SU5416 (+SU) and with EPC (+SU + EPC). Treatment with SU5416 results in loss of *Pecam* expression in SU-treated explants (+SU). Addition of EPC (+SU + EPC) does not restore *Pecam* expression. Increased acinar differentiation (*Cpa* and *Amy* expression) is observed upon treatment with SU5416 (+SU) for 2 and 3 days of culture. On the contrary, addition of exogenous endothelial cells (+SU + EPC) blocks the induction of acinar differentiation markers. (Mann-Whitney for control explants: °p < 0.05; and for SU- and SU + EPC-treated explants: *p < 0.05; **p < 0.01). (**b**) PCR for the endothelial marker *Cdh5* (174 bp) in control explants, explants treated with SU5416 (+SU) and explants treated with SU5416 and EPC (+SU + EPC) for 3 days. *Cdh5* is expressed in control explants, but is absent from SU5416-treated explants (+SU). Addition of EPC on endothelium-deprived explants (+SU + EPC) results in the restoration of *Cdh5* amplicon. • = non-specific amplicons. (**c**) Phase-contrast image of pancreatic explants cultured for 3 days (D3) on filters. Exogenous endothelial cells were added on SU5416-treated pancreatic explants (+SU + EPC) or not (Ctrl). EPC remained clustered around the explants during the culture.

In developing thyroid, we have shown that embryonic endothelial progenitor cells (EPC) can replace blood vessels and stimulate follicular lumen expansion^14,15^. To test the possibility that EPC could also functionally replace endothelial cells in developing pancreatic explants, we first depleted endogenous endothelial cells by culturing explants with the VEGFR2 inhibitor SU5416 (+SU) for 2 or 3 days. We have previously reported that this treatment rapidly induces apoptosis of endothelial cells without affecting survival of the pancreatic epithelium^12^. Efficacy of endothelial depletion was assessed by measuring the expression of the endothelial cell marker *Pecam*. RT-qPCR revealed that within 2 days of culture in the presence of SU5416, *Pecam* expression was dramatically decreased (Fig. 2a, grey bars). In these endothelium-free explants, we next investigated the expression of the acinar markers *Cpa* and *Amy*. Expression of both genes was increased after 2 and 3 days of culture in the presence of SU5416 (+SU) confirming increased acinar differentiation in the absence of endothelial cells (Fig. 2a), as reported^12^. To evaluate whether addition of cultured endothelial cells can functionally replace endogenous ones, and thus reverse the increase in acinar differentiation, we supplemented SU-treated explants with 50,000 EPC (+SU + EPC). EPC added on the culture filter remained clustered and closely associated with the pancreatic explants (Fig. 2c). *Pecam* expression level in +SU + EPC samples did not change, as EPC cells do not express *Pecam* (Fig. 2a, dark grey bars). However, PCR analysis revealed that addition of 50.000 EPC to SU-treated pancreatic explants restored Cdh5 expression level to control levels, and further indicated that EPC remain alive during the culture with the explants (Fig. 2b). In line with our hypothesis, addition of EPC to SU-treated explants blocked the induction of the acinar markers *Cpa* and *Amy*. Expression of *Cpa* and A*my* were similar in +SU + EPC and control explants at 2 days, and did not increase at 3 days (Fig. 2a, dark grey bars). This result indicates that exogenous EPC not only replaced endogenous endothelial cells but also prevented normal acinar differentiation *ex vivo*.

### Endothelial progenitor cells limit acinar differentiation via a soluble factor

**Figure 3:**
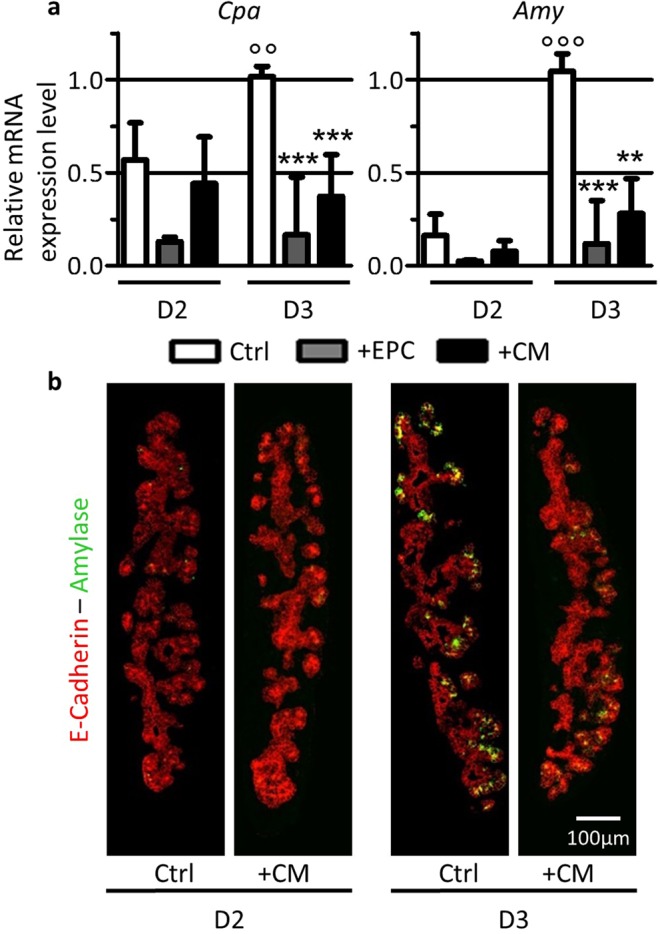
Medium conditioned by EPC limits acinar differentiation. (**a**) RT-qPCR analysis of acinar markers *Cpa* and *Amy* reported to *β-actin* in explants cultured for 2 and 3 days in the presence of control medium (Ctrl), endothelial cells (+EPC) or of medium conditioned by EPC (+CM). Both EPC and CM limit acinar differentiation. (Mann-Whitney for control explants: °°p < 0.01; °°°p < 0.001; and for EPC- and CM-treated explants: **p < 0.01; ***p < 0.001). (**b**) Immunofluorescence for Amylase (green) within the pancreatic epithelium (E-Cadherin, red) of control explants (Ctrl) or of explants treated with conditioned medium (+CM) at 2 and 3 days. Fewer Amylase^+^ cells are observed following culture with CM.

We next investigated whether the repressive effect of endothelial cells on acinar differentiation was exerted by cell-cell contact with the pancreatic epithelium or by the release of one or several endothelial-derived factor(s). Therefore, pancreatic explants were cultured in the presence of endothelial cells (+EPC) or medium conditioned by EPC (+CM), and expression of acinar markers was analyzed (Fig. 3a). Although addition of EPC or CM to pancreatic explants affected *Cpa* and *Amy* expression from the second day of culture, the effect was more pronounced and significant after 3 days. Both EPC and CM blocked *Cpa* and *Amy* induction. The effect of CM on acinar differentiation was also observed at the protein level on tissue sections (Fig. 3b). Immunofluorescence analysis of Amylase (green) revealed the presence of few Amylase^+^ cells in the pancreatic epithelium, labeled for E-Cadherin (red), after 2 days in culture. After 3 days in culture, the number of Amylase-expressing cells was considerably increased, mainly at the extremities of epithelial branches; a location compatible with developing acinar structure from tip cells. As observed by RT-qPCR, addition of CM to the explants reduced the number of Amylase^+^ cells. Altogether, these experiments indicate that EPC prevent acinar differentiation via the release of soluble factor(s) in the CM.

### Medium conditioned by EPC prevents the expression of members of the PTF1L acinar complex

**Figure 4:**
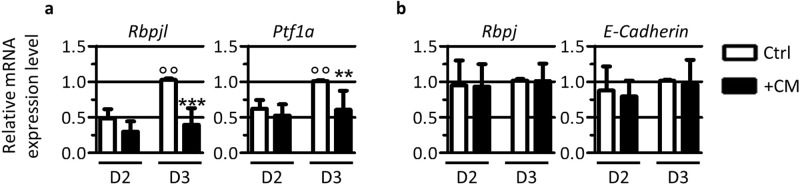
EPC-CM prevents the formation of the acinar PTF1L-complex. (**a**) RT-qPCR analysis of members of the PTF1L-complex *Rbpjl* and *Ptf1a* reported to *β-actin* in explants cultured for 2 and 3 days with control medium (Ctrl) or EPC-CM (+CM). In control explants, expression of pro-acinar transcription factor *Rbpjl* and of *Ptf1a* is induced from 2 to 3 days of culture. EPC-CM blocks the induction of *Rbpjl* and *Ptf1a* in developing explants. (**b**) RT-qPCR analysis of *Rbpj* and *E-Cadherin* reported to *β-actin* in the same culture conditions reveals that CM does not globally affect gene transcription. (Mann-Whitney for control explants: °°p < 0.01; and for EPC-CM-treated explants: **p < 0.01; ***p < 0.001).

Acinar differentiation is initiated by a switch in the PTF1 transcriptional complex. In acinar progenitor cells, the transcription factors RBPJ and PTF1A associate with a small bHLH protein to form the trimeric PTF1J-complex. This trimeric unit regulates the expression of genes maintaining cells in an undifferentiated state. PTF1J-complex also induces the expression of *Rbpjl*, resulting in the accumulation of the protein that progressively replaces RBPJ within the trimeric PTF1-complex to form the PTF1L-complex. This PTF1L-complex induces the expression of acinar genes like *Amy* and is thus considered as a pro-acinar transcriptional complex^3,4^. As both complexes, PTF1J and PTF1L, also induce the expression of *Ptf1a*, the level of this latter increases during this period of pancreas development. We first verified that the PTF1J to PTF1L switch occurred in cultured pancreatic explants. By RT-qPCR we measured the expression of the pro-acinar component of the PTF1L trimeric complex, *Rbpjl* and of *Ptf1a* (Fig. 4a). We also measured the expression of *Rbpj* and *E-Cadherin*, as controls (Fig. 4b). In untreated explants, we observed a ± 2-fold increase in the expression of the pro-acinar subunit *Rbpjl* and of *Ptf1a* from D2 to D3 of culture (Fig. 4a). These results are in agreement with the increase of acinar markers *Cpa* and A*my* over the same period of culture (Fig. 1b). Expression of the control genes *Rbpj* and *E-Cadherin* did not change over the same period (Fig. 4b). We next tested the effect of EPC-CM on the expression of PTF1L pro-acinar genes. Although we could only observe a slight decrease in *Rbpjl* and *Ptf1a* expression after 2 days, the effect of EPC-CM was significant after 3 days (Fig. 4a). Pancreatic explants cultured in the presence of CM showed no induction of *Rbpjl* and *Ptf1a* genes, whose levels remained similar to those observed at 2 days in the control condition. Expression of *Rbpj* and *E-Cadherin* was not affected by EPC-CM, indicating that CM specifically represses the expression of the pro-acinar components of the mature PTF1L-complex and thus acinar differentiation.

### EPC-CM contains SPARC and laminin-α1β1γ1 that regulate acinar differentiation

**Figure 5:**
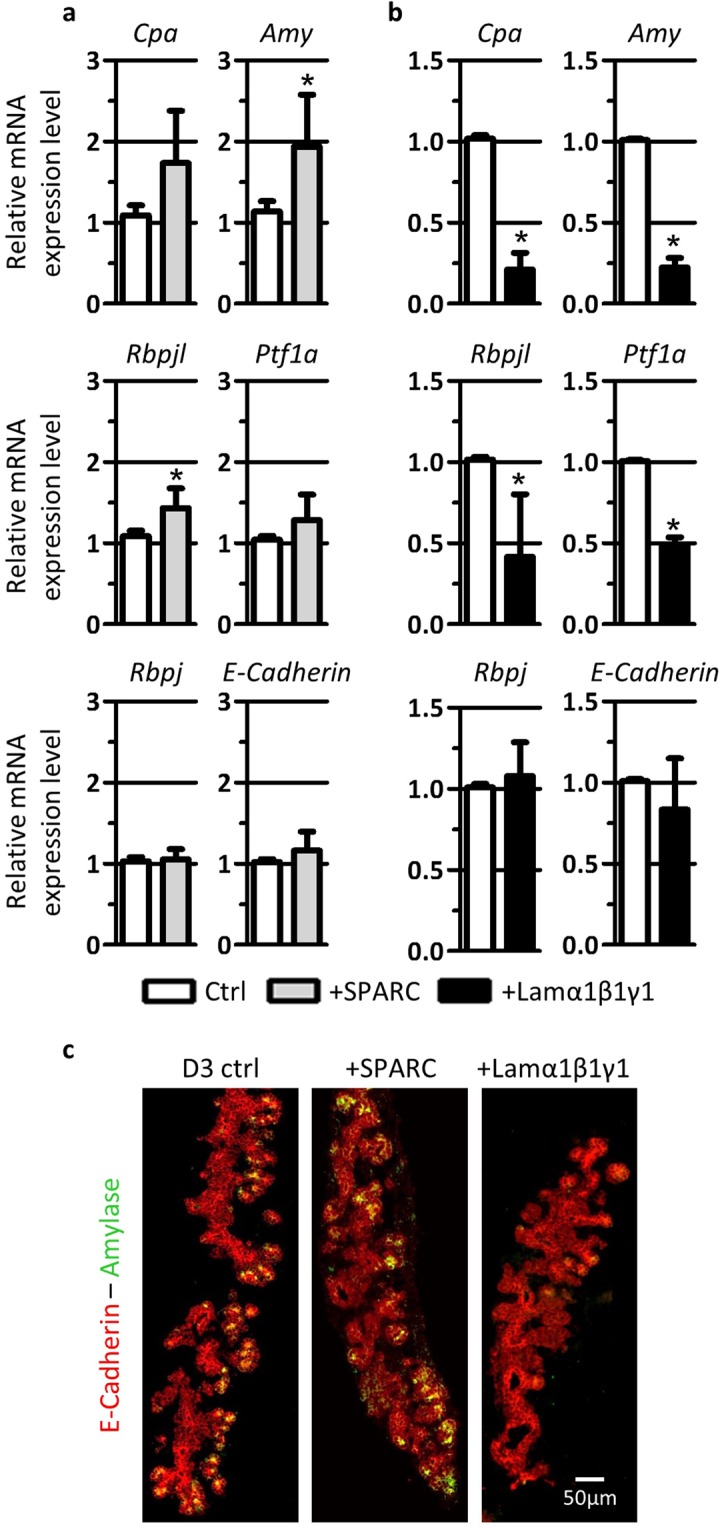
SPARC and laminin-α1β1γ1, two proteins abundantly found in EPC-CM, control acinar differentiation. (**a**) RT-qPCR analysis of acinar and control genes reported to *β-actin* in pancreatic explants cultured for 3 days in control medium (Ctrl) and in the presence of SPARC (+SPARC). Addition of SPARC favors acinar differentiation as demonstrated by increased levels of acinar markers (*Cpa* and *Amy*) and pro-acinar transcription factors (*Rbpjl* and *Ptf1a*). Expression level of control genes (*Rbpj* and *E-Cadherin*) are not affected. (**b**) RT-qPCR analysis of acinar and control genes reported to *β-actin* in pancreatic explants cultured for 3 days in control medium (Ctrl) and in the presence of laminin-α1β1γ1. In the presence of laminin-α1β1γ1, the expression of acinar markers (*Cpa* and *Amy*) and pro-acinar transcription factors (*Rbpjl* and *Ptf1a*) is reduced as compared to control explants. Expression level of control genes (*Rbpjl* and *E-Cadherin*) is not affected. (Mann-Whitney: *p < 0.05). (**c**) Immunofluorescence for the acinar differentiation marker Amylase (green) and the pancreatic epithelium marker (E-Cadherin, red) in explants at 3 days, as indicated. As compared to control explants showing acinar differentiation (Amylase^+^ cells), addition of SPARC stimulates, while laminin-α1β1γ1 decreases the Amylase signal.

Mass spectrometry analysis of EPC-CM revealed that secreted protein acidic and cysteine rich (SPARC) and laminin-α1β1γ1 are the most abundant proteins released by EPC^15^. SPARC is known to play a role in the regulation of the assembly of the extracellular-matrix (ECM), and laminins are self-assembling macromolecules of the ECM, more specifically of the basement membrane^16,17^. We tested whether addition of SPARC or of purified laminin-α1β1γ1 on cultured pancreatic explants could mimic the limiting effect of EPC-CM on acinar differentiation. Based on the literature, we tested two concentrations of recombinant mouse SPARC protein (1 µg/ml and 5 µg/ml) in pancreatic explant cultures^18^. As both concentrations gave similar results, we performed most experiments at the lowest concentration (1 µg/ml), corresponding to the manufacturer’s recommendations. Upon incubation of pancreatic explants with SPARC for 3 days, we found that the expression of the acinar markers (*Cpa* and A*my*), and of the pro-acinar components of the PTF1L-complex (*Rbpjl* and *Ptf1a*) were not decreased, as expected for an anti-acinar factor. On the contrary, expression of *Amy* and *Rbpjl* were significantly increased (Fig. 5a). Addition of SPARC did not modify the expression of the control genes, *Rbpj* and *E-Cadherin*. Immunohistological analysis confirmed that the Amylase signal was stronger in pancreatic explants cultured in the presence of recombinant SPARC (Fig. 5c). We concluded that recombinant SPARC used on its own does not behave as an anti-acinar factor, as it positively controls the expression of some pancreatic acinar differentiation genes *ex vivo*.

Incubation of pancreatic explants with purified laminin-α1β1γ1 dose-dependently affected epithelial growth and morphogenesis at the higher concentrations tested (4.5 and 15 µg/ml, data not shown). We thus used a working concentration of laminin-α1β1γ1 (2.5 μg/ml) at which no morphological effect could be observed and the expression level of *E-Cadherin*, used as a sensitive readout of epithelial changes, was comparable to that of untreated explants. At this optimized concentration, purified laminin-α1β1γ1 caused a significant decrease in the expression of *Cpa* and A*my*, as well as of *Rbpjl* and *Ptf1a* (Fig. 5b). It is interesting to note that the repressive effect of laminin-α1β1γ1 on these 4 genes was comparable to the effect of the EPC-CM (compare Fig. 5b with Figs 3a and 4a). Expression of *Rbpj* remained unaffected by the addition of exogenous laminin-α1β1γ1. These observations were confirmed by immunofluorescence analysis of pancreatic explants cultured with purified laminin-α1β1γ1 (Fig. 5c). As compared to control explants, those cultured in the presence of laminin-α1β1γ1 showed almost no signal for the acinar marker Amylase within the pancreatic epithelium. These results indicate that purified laminin-α1β1γ1 represses acinar differentiation in cultured pancreatic explants.

### EPC-CM might promote ductal cell differentiation

**Figure 6:**
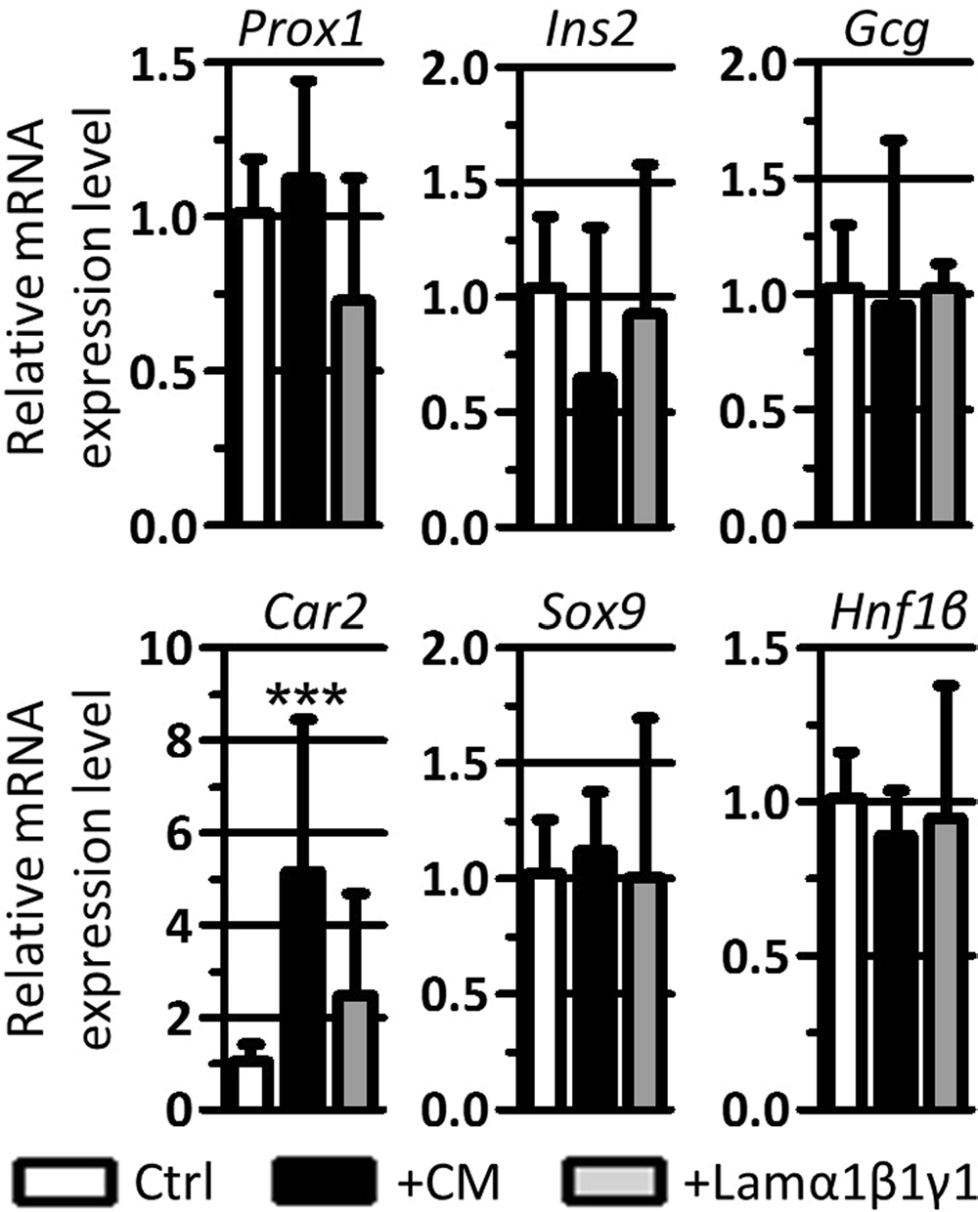
EPC-CM treatment induces the expression of Carbonic Anhydrase 2 (*Car2*). RT-qPCR analysis of progenitor, endocrine and ductal markers reported to β-actin in pancreatic explants cultured for 3 days in control medium (Ctrl), EPC-CM (+CM) and in the presence of laminin-α1β1γ1 (+Lamα1β1γ1). EPC-CM and laminin-α1β1γ1 do not affect expression of progenitor (*Prox1*) and endocrine (*Ins2* and *Gcg*) markers. Conversely, the treatments upregulate the expression of the ductal marker *Car2*, although the expression of the ductal transcription factors *Sox9* and *Hnf1β* remain stable. (Mann-Whitney: ***p < 0.001).

Explants treated with either EPC-CM or purified laminin-α1β1γ1 showed unaltered expression of *E-Cadherin* (Figs 4 and 5b), suggesting that these treatments do not influence the abundance of epithelial cells. Since addition of EPC-CM or laminin-α1β1γ1 blocks acinar differentiation, we wondered whether the acinar progenitors remained in an undifferentiated state or switched to another pancreatic cell differentiation program. The expression of the endodermal marker *Prox1* and the endocrine markers Insulin (*Ins2*) and Glucagon (*Gcg*) were not affected by EPC-CM or laminin-α1βγ addition (Fig. 6). Moreover, immunolocalization of insulin in control, CM- and laminin-α1β1γ1-treated explants showed no differences in the abundance and localization of insulin-expressing cells (data not shown).

On the contrary, expression of Carbonic Anhydrase 2 (*Car2*), a differentiated ductal cell marker, was highly upregulated in the presence of EPC-CM, but not statistically different in the presence of laminin-α1β1γ1. Surprisingly, *Car2* induction by EPC-CM was not accompanied by an increased expression of the ductal transcription factors *Sox9* and *Hnf1β*, thereby suggesting a differentiation effect of EPC-CM rather than an increase in the number of ductal cells. Altogether, we conclude that addition of EPC-CM to pancreatic explants seems to favor ductal cell differentiation, while blocking acinar differentiation.

### Laminin-α1 is present in developing pancreas but not in the vicinity of developing acini

**Figure 7:**
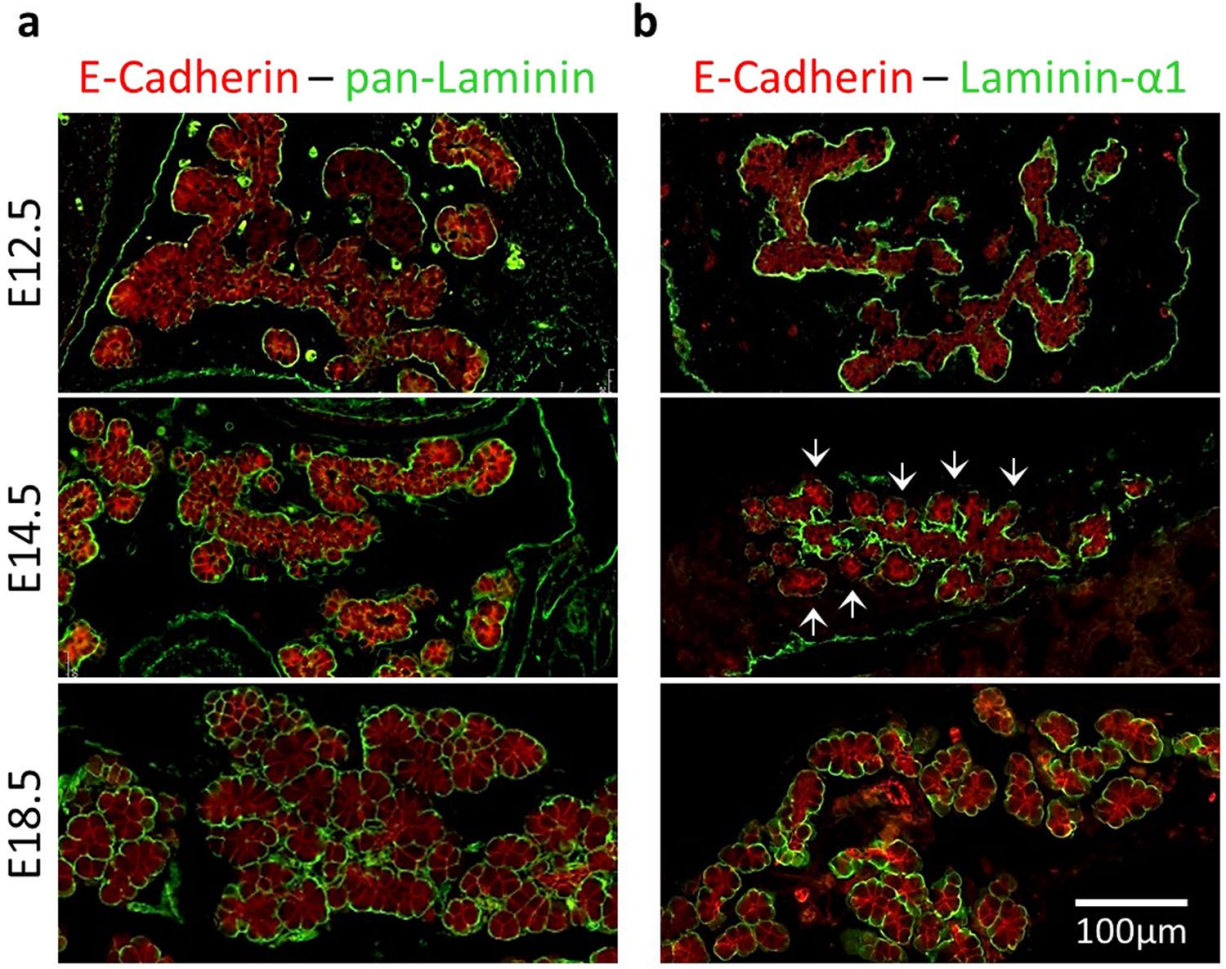
Differential laminin-α1 deposition during pancreas development *in vivo*. (**a**) Immunolabeling for pan-Laminin (green) with the epithelial markers (E-Cadherin, red) in pancreas at E12.5, E14.5 and E18.5. At E12.5, pan-Laminin antibody recognizes an almost continuous structure around the branching pancreatic epithelium. At E14.5 and E18.5, the pan-Laminin signal homogenously surrounds the differentiating pancreatic epithelium. (**b**) Immunolabeling for laminin-α1 (green) with the epithelial marker (E-Cadherin, red) in developing pancreas (E12.5-E14.5-E18.5). Laminin-α1 antibody recognizes the same structures as pan-Laminin, located around the branching pancreatic epithelium. However, at E14.5, the laminin-α1 signal is much stronger around the trunk cells than around the tip cells (arrows). This is transitory as at E18.5, laminin-α1 is homogenously found around the pancreas.

As our *ex vivo* results indicate that laminin-α1β1γ1 behave as an anti-acinar factor, we wanted to verify whether distribution of this laminin *in vivo* could be compatible with such an effect. Immunolocalization with a pan-Laminin antibody revealed a continuous basal lamina separating the epithelial cells from the stroma, at all stages of *in vivo* pancreas development (Fig. 7a). On the contrary, laminin-α1 immunolabeling showed a more subtle and dynamic pattern. During the early stages of pancreas organogenesis, at E12.5, laminin-α1 was detected all around the developing pancreas, separating the epithelial cells from the surrounding mesenchyme (Fig. 7b). Interestingly, at E14.5, laminin-α1 distribution was more heterogeneous. Laminin-α1 was detected mainly around the pancreatic trunk and branches, and to a lesser extent around developing acini where the signal was weak and discontinuous (Fig. 7b, arrows). This particular pattern was transient and before birth (E18.5), laminin-α1 was again delineating all the pancreatic epithelial cells, even the differentiated acinar pyramidal cells. These data indicate that during acinar differentiation, laminin-α1 is not present uniformly around all the epithelial cells of the developing pancreas but is predominantly localized around trunk cells and reduced around the growing tip cells undergoing acinar differentiation. This laminin-α1 localization pattern is highly reminiscent to the localization of blood vessels previously described^12^. Altogether, these data suggest that differential expression or deposition of laminin-α1 could regulate pancreatic acinar differentiation.

## Discussion

In this work, we used a validated *ex vivo* culture system of embryonic pancreata, micro-dissected at embryonic (E) day 12.5, to study the control of acinar differentiation by extrinsic signals. Based on our results, we propose a model in which endothelial cells would promote, directly or indirectly, deposition of laminin-α1β1γ1 locally around pancreatic trunk cells, at a distance from the acinar progenitors or tip cells. The presence of laminin-α1β1γ1 would prevent the expression of the main components of the pro-acinar transcriptional complex PTF1L (namely *Rbpjl* and *Ptf1a*) in the pancreatic trunk cells, and in consequence, of the acinar genes *Cpa* and *Amy*, thus controlling acinar differentiation. Tip cells, localized at a distance from endothelial cells and of laminin-α1β1γ1 would turn on the acinar differentiation program.

Several groups have already used e*x vivo* culture systems to decipher mechanisms of pancreas development and differentiation^13,19,20^. Indeed, *ex vivo* pancreas culture not only recapitulates pancreatic growth, branching and differentiation, but also allows experimental manipulation difficult to perform *in vivo*. Here, we submitted pancreatic explant cultures to pharmacological ablation of endothelial cells (SU5416) and exogenous addition of endothelial cells, of their conditioned medium, and of purified or recombinant proteins. At appropriate time points, gene and protein expression patterns were analyzed from the cultured pancreatic explants. Explant culture is thus a powerful system to decipher mechanisms controlling organogenesis.

Endothelial cell ablation in SU5416-treated explants resulted in excessive acinar differentiation, as previously shown^12^. This could be reversed by addition of exogenous endothelial progenitor cells (EPC). These cells were chosen because of their demonstrated role in embryonic thyroid follicle formation^14,15^. Moreover, PAE (Porcine Aortic Endothelial cells) and HUVEC (Human Umbilical Vein Endothelial Cells) did not show any anti-acinar effect (data not shown). Three different amounts (30,000, 50,000 and 75,000 EPC) were initially tested and were found to prevent acinar differentiation. In all our experiments, we decided to add 50,000 EPC on top of each filter containing up to 4 pancreatic explants. EPC remained in aggregates in the vicinity and in contact with the periphery of the explants. As we did not observe deep EPC invasion into the explants, we assumed that endothelial repression of acinar differentiation was mediated by the release of a factor from adjacent EPCs rather than by epithelial-endothelial contacts. This was indeed confirmed by addition of medium conditioned by EPC (EPC-CM). Although, we decided to work with a 10-fold concentrated EPC-CM as in our thyroid studies^15^, it is worth mentioning that the effect of EPC-CM on acinar differentiation was strictly concentration-dependent, and effects started to be observed with a 3-fold concentrated EPC-CM. Requirement to concentrate EPC-CM might be necessary in our experimental setting to compensate the *in vivo* local effect of bioactive factor(s).

It is largely accepted that endothelial cells play an important role in organ development, growth and differentiation. Besides their role as building blocks of nutritive pipes, they also produce various factors influencing their environment^21,22^. Recently, our laboratory provided evidence that thyroid folliculogenesis requires the presence of endothelial cells, and that this process can be stimulated *ex vivo* by EPC-CM and EPC-derived microvesicles^15,23^. Our results now show that EPC-CM-treated pancreatic explants display decreased acinar differentiation, and increased ductal differentiation, as compared to control explants. We ruled out that that EPC-CM stimulates apoptosis of acinar cell progenitors, or inhibits their proliferation by immunolabeling explants with activated Caspase-3 and phospho-histone H3 antibodies (data not shown). We thus focused on acinar differentiation. The molecular mechanism triggering acinar differentiation has been well studied by the group of Mac Donald^3,4^. Around embryonic day E14.5, RBPJ is replaced by RPBJL within the trimeric transcriptional PTF1J-complex to form the pro-acinar PTF1L-complex. The latter regulates the expression of acinar genes coding for digestive enzymes. Our results showed that EPC-CM prevents the induction of two components, *Ptf1a* and *Rbpjl*, of the pro-acinar PTF1L-complex. Regulation of *Rbpjl* expression is not yet fully understood. Previous work has shown that its expression is initiated by the PTF1J-complex, containing PTF1A. Furthermore, *Ptf1a* expression is controlled by the PTF1L-complex, containing RBPJL and PTF1A^3,4^. Therefore, it is difficult to precisely identify the mechanisms by which EPC-CM limit acinar differentiation. Nevertheless, we predict that EPC-CM might predominantly act on the expression of *Ptf1a*. Three separable regulatory regions have been shown to control temporal and spatial *Ptf1a* expression^24^. The promoter region, upstream of the transcription initiation site, is responsible for basal transcription. A second region localized downstream of the last exon of *Ptf1a* directs expression in the dorsal part of the spinal cord and has limited role in the embryonic pancreas. A third distal upstream enhancer complements the activity of the promoter region. During pancreas development, this enhancer superinduces *Ptf1a* expression in the acinar cells at the onset of their development via conserved binding sites for the PTF1L-complex. These binding sites are also necessary for *Ptf1a* gene expression maintenance^24,25^. Thus, EPC-CM might target and inhibit *Ptf1a* regulatory regions by regulating transcription factors or chromatin remodeling factors. The molecular mechanisms by which extrinsic factors control acinar differentiation and the PTF1 transcriptional complexes are poorly understood, despite evidences for the involvement of these extrinsic factors. Indeed, several groups have proposed that either mesenchymal FGF-10 or signals from aortic endothelial cells are required for early induction of *Ptf1a* gene expression^7,26^, but the mechanism has not been elucidated. Our results further indicate that regulation of acinar differentiation and of PTF1L-complex can be controlled, directly or indirectly, by endothelial-derived molecules contained in EPC-CM.

To understand how EPC limit expression of *Ptf1a* and *Rbpjl*, and thus acinar differentiation, we focused on SPARC and laminin-α1β1γ1, previously identified by mass-spectrometry in EPC-CM^15^. It has been shown that endothelial cells produce SPARC, which, in turn, influences the stability of the extracellular matrix, and thus regulate endothelial attachment and proliferation^27,28^. These data support the idea that SPARC is an anti-angiogenic factor and, according to our model, would behave as a pro-acinar factor in pancreatic explants. This was indeed the case, as expression of *Amy* and *Rbpjl* were increased in explants cultured in the presence of exogenous SPARC. However, expression levels of endothelial markers *Pecam*, *Cdh5* and *Flk-1* were stable in SPARC-treated explants as compared to control explants (data not shown).

SPARC has also been shown to regulate basal lamina assembly. Basal lamina is mainly composed of collagen IV and laminins. A study in *Caernorhabditis elegans* showed that overexpression of SPARC led to altered collagen IV trafficking and decreased incorporation of this protein into the basement membrane^29^. Another group studied the role of SPARC in the regulation of lens epithelium basement membrane assembly. Correct basal lamina composition and assembly is required for normal morphology, differentiation and function of the lens epithelial cells^30^. This study first showed that SPARC and laminin-α1β1γ1 need to be co-secreted in order for laminin-α1β1γ1 to be correctly assembled into the basement membrane. Secondly, they showed that, in SPARC knockout mice, excessive laminin-α1β1γ1 was deposited and formed aggregates around epithelial cells, which fail to differentiate correctly. Thus, SPARC is an important regulator of basement membrane formation. In pancreatic explants, we found that SPARC is predominantly present in epithelial tip and acinar cells (Suppl. Fig. S2). This acinar localization confirms data from The Human Protein Atlas database^31^, and is entirely compatible with a pro-acinar function. One can postulate that, at E14.5, in the absence of endothelial cells, there is no laminin-α1β1γ1 and thus no effect of SPARC, but later on, SPARC and laminin laminin-α1β1γ1 might further drive acinar differentiation.

Another possibility would be that SPARC regulates the expression and/or activity of matrix metalloproteinases (MMPs) locally. Indeed, several groups have reported a positive correlation between SPARC expression and presence of MMPs. More specifically, they showed that MMP-2 expression can be regulated by SPARC^32,33^. Another study identified laminin-α1β1γ1 as a substrate of MMP2^34^. We therefore do not exclude the possibility that, in our model, SPARC could regulate the expression and/or activity of MMP-2 around developing acini, and thus control differential degradation of laminin-α1β1γ1 in order to stimulate acinar differentiation.

Mass spectrometry analysis of EPC-CM also revealed abundant peptides from laminin chains α1, β1 and γ1^15^. Laminins are heterotrimeric molecules resulting from the assembly of an α-, a β- and a γ-chain to form at least 15 different heterotrimers, in mammals. By interacting with ECM macromolecules such as collagens, and cell-surface molecules such as integrins, laminins fulfill structure-forming and cell-adhesive functions. They play essential roles in organogenesis, growth and differentiation of lung, mammary gland, thyroid and other organs^15,35,36^. In the pancreas, endocrine differentiation depends on the deposition of laminins by endothelial cells. By interacting with β1-integrins, laminins signal to β-cells and stimulate insulin expression^9,37^. Moreover, pancreatic acinar cells of laminin-α2 and -α4 dKO mice fail to assemble a basement membrane, and this affects cell polarization and development^38^. The group of Gittes further showed that *ex vivo* initiation of exocrine differentiation and ductal morphogenesis require interaction of laminin-α1β1γ1 with integrin-α6β1^37^. Thus, different laminin isoforms seem to play important roles at various stages of pancreas development. Addition of purified laminin-α1β1γ1 on pancreatic explants prevented acinar differentiation, thereby mimicking the effect of EPC and EPC-CM. We thus postulate that EPC and EPC-CM prevent acinar differentiation by production and deposition of excessive amount of laminin-α1β1γ1 around the pancreatic epithelial cells of the explants, and thus around tip cells. Presence of exogenous laminin-α1β1γ1 around growing tip cells would prevent the induction of *Ptf1a* and *Rbpjl*, and thus of the acinar differentiation program. This hypothesis is supported by the particular localization of laminin-α1 in developing pancreas. At E14.5, corresponding to the onset of acinar differentiation, laminin-α1-containing heterotrimers are predominantly found around the ductal cells, at a distance from developing acini. This observation confirms published studies on human fetal pancreatic sections where laminin-α1 is predominantly found around ductal structures^39^. We therefore propose that temporal and spatial regulation of laminin-α1β1γ1 deposition, possibly through localized SPARC co-secretion, controls adequate ductal and acinar pancreas development. Laminin-α1β1γ1 would repress the expression of two components of the pro-acinar transcriptional PTF1L-complex, in the trunk cells. Conversely, in the tip cells, not exposed to abundant basal laminin-α1β1γ1, acinar differentiation would be initiated.

Based on our previous work, showing that endothelial cells repress acinar differentiation during pancreas development, we here further show that acinar differentiation can be controlled by differential laminin-α1β1γ1 deposition around the epithelial trunk cells, at a distance from the differentiating acinar cells. Indeed, our data indicate that laminin-α1β1γ1 is an anti-acinar factor that negatively regulates the expression of two components of the pro-acinar transcriptional PTF1L-complex.

## Methods

### Animals

Pdx1-GFP mice were obtained from D.Melton^40^. All other mice were of the CD1 strain. The animals were raised and treated according to the NIH Guide for Care and Use of Laboratory Animals, and experiments were approved by the University Animal Welfare Committee, Université Catholique de Louvain (2016/UCL/MD/005). Females were mated and the day of the vaginal plug was considered as embryonic day (E) 0.5. Pregnant females were sacrificed by cervical dislocation at E12.5.

### Embryonic endothelial progenitor cells (EPCs)

EPCs were cultured on gelatin-coated T75 culture flasks in DMEM (Westburg) containing 20% FBS, 100 U/mL penicillin, 100 µg/mL streptomycin and 100 µM non-essential amino acids, as described^41,42^. Cell passaging was performed using Tryple Express (Invitrogen).

Conditioned medium (CM) was prepared as described^15^. Briefly, 6 ml of M199 medium supplemented with 100U/ml penicillin, 0.25 µg/ml fungizone, 100 µg/ml streptomycin and 2 mM glutamine was added to 80% confluent EPCs in 78 cm² dish. After 24 h, CM was collected, centrifuged for 5 min at 1,900 g to eliminate debris and dead cells, and the supernatant was further spun for 20 min at 17,000 g. Finally, CM was concentrated 10X using Amicon Ultra 50 K unit filters for ±7 min at 1,900 g.

### Pancreatic explant dissection and culture

E12.5 pancreatic dorsal buds were microdissected and up to 4 explants were placed on a microporous filter (Millipore) resting on M199 medium (Invitrogen) supplemented with 10% FBS, 100U/ml penicillin, 0.25 µg/ml fungizone, 100 µg/ml streptomycin and 2 mM glutamine^14^ (control condition). Medium was supplemented with either 1 µg/ml recombinant mouse SPARC (R&D Systems), 2.5 µg/ml purified laminin-α1β1γ1 (gift from T. Sasaki), 3 µM of VEGFR2 inhibitor (SU5416, VWR), or vehicles. For EPC treatment, 50,000 cells were added on top of the filter, in contact with the explants. For culture with conditioned medium (CM) from EPC, M199 was replaced by EPC-CM. For all culture, medium was renewed at mid-term culture, and 10 µl from the culture medium were added on top of the filter 3 times/day.

### RT-qPCR

Total RNA was extracted from cultured pancreatic explants using TRIzol Reagent (Thermo Scientific), as described^43^. Reverse transcription was performed on 500 ng of total RNA using M-MLV Reverse Transcriptase (Invitrogen) and random hexamers. Real-time quantitative PCR was carried out on cDNA samples using the KAPA SYBR Fast qPCR kit (Sopachem) according to the manufacturer’s instructions. Primers sequences are listed in Table S1. Data were analyzed using the ΔΔCT method, using *β-Actin* as reference gene^44^.

### Immunofluorescence on gelatin sections

Pancreatic explants were fixed in 4% formaldehyde for 1 h, incubated overnight at 4 °C with 20% sucrose in PBS and embedded in PBS/15% sucrose/7.5% gelatin. Sections (7 µm), obtained with a cryostat (CryoStar NX70, ThermoScientific), were immersed in warm PBS (40 °C) for 5 min to remove gelatin or in citrate buffer (10 mM, pH 6.0) heated 2 × 5 min in a microwave (750Watt). After permeabilization in PBS/0.3% Triton X-100 for 5 min, non-specific sites were blocked by 45 min incubation with PBS/0.3% Triton X-100/10% Bovine Serum Albumin (BSA)/3% milk (blocking solution). Primary antibodies (Table S2), diluted in blocking solution, were incubated overnight at 4 °C. After washing in PBS/0.1% Triton X-100, secondary antibodies coupled to Alexa-488, -568 or -647 (Invitrogen) and fluorescent nuclear dye (Hoechst 33258; Sigma) were diluted (1/2,000) in PBS/10% BSA/0.3% Triton X-100, and incubated for 1 h at room temperature. After extensive washing, slides were mounted using aqueous Dako Mounting Medium and analyzed with a Zeiss Cell Observer Spinning Disk confocal microscope or Pannoramic P250 Digital Slide Scanner.

### Immunofluorescence on paraffin sections

The caudal half of E12.5, E14.5 and E18.5 CD1 embryos were fixed in 4% formaldehyde for 1 h and embedded in paraffin using a Tissue-Tek VIP-6 (Sakura). Sections of 7 µm were obtained with the microtome Micron HM355S (ThermoScientific). After paraffin removal, slides were treated similarly as gelatin sections.

### Whole-mount immunolabeling

For 3D analysis, pancreatic explants were fixed in 4% formaldehyde for 1 h and transferred into TBST (50 mM Tris HCl pH7.5, 150 mM NaCl, 0.1% Triton X-100). Blocking was performed in TBST containing 10% normal goat serum (NGS) for 45 min at room temperature. Primary antibodies were diluted in blocking buffer (Table S2), and incubated overnight at 4 °C. The explants were washed thoroughly (minimum 5 one-hour washes) and incubated with secondary antibodies coupled to Alexa-488 or -568 (1/500) diluted in TBST/1% NGS, overnight at 4 °C. After repeated washing (minimum 5 one-hour washes), explants were post-fixed in 4% formaldehyde during 10 min and left in TBST until observation or tissue clearing.

For tissue clearing, immunolabeled pancreatic samples were dehydrated in increasing concentration (up to 100%) of methanol. Then, methanol was replaced by methyl salicylate by progressive addition of methyl salicylate (10% increment/30 min). Cleared samples were finally placed on a round coverslip for z-stack imaging with a Zeiss Cell Observer Spinning Disk confocal microscope.

### Statistical analysis

All RT-qPCR values were obtained by the ΔΔCT method and are expressed as means ± standard deviation (SD). Each graph represents minimum 3 independent experiments on minimum 3 pancreatic explants. Nonparametric statistical tests were used: Mann-Whitney for comparison of 2 conditions and Kruskal-Wallis followed by Dunn’s post-test for more conditions. Differences were considered statistically significant when p < 0.05. For control conditions over time: °stands for p < 0.05; °°for p < 0.01; °°°for p < 0.01. For control condition versus experimental condition: *stands for p < 0.05; **for p < 0.01; ***for p < 0.01.

## Supplementary information

Supplementary Dataset 1

## Data Availability

Materials, data and associated protocols are available.
