# Supplementary material for: Pancreatic acinar differentiation is guided by differential laminin deposition
Source: Sci Rep. 2019 Feb 25;9:2711. doi: 10.1038/s41598-019-39077-6 (PMC6389953; doi:10.1038/s41598-019-39077-6)
Supplement: Supplementary file 1 — Supplementary Dataset 1 [file 41598_2019_39077_MOESM1_ESM.pdf]

Pancreatic acinar differentiation is guided by differential laminin deposition.

Charlotte Heymans, Jonathan Degosserie, Catherine Spourquet & Christophe E. Pierreux

Supplementary Information

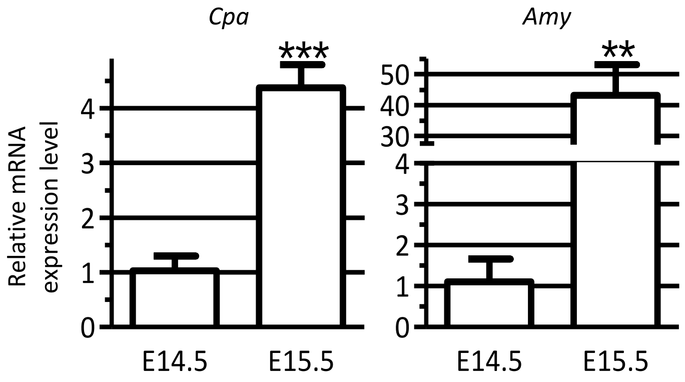

Figure S1: Acinar differentiation increases from E14.5 to E15.5 *in vivo*.
RT-qPCR analysis of acinar markers *Cpa* and *Amy* compared to *β-actin* on E14.5 and E15.5 pancreata. The increase of *Cpa* and *Amy* expression illustrates acinar differentiation. (Student T-test: **, $p<0.01$; ***, $p<0.001$).

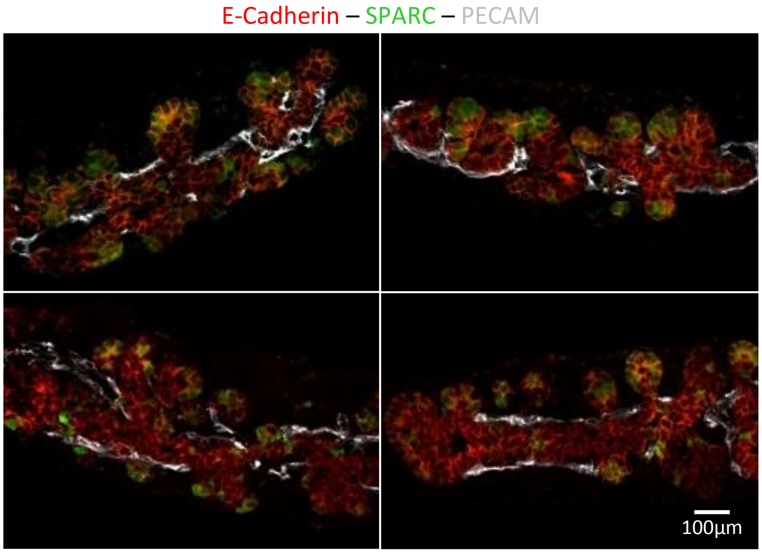

Figure S2: SPARC is found in developing acini.
Immunofluorescence for SPARC (green), the pancreatic epithelium marker (E-cadherin, red) and the endothelial marker PECAM (white) in explants at 3 days. SPARC is predominantly found in the epithelial cells located at the periphery of the explant, at a distance of endothelial cells (PECAM$^+$ cells).

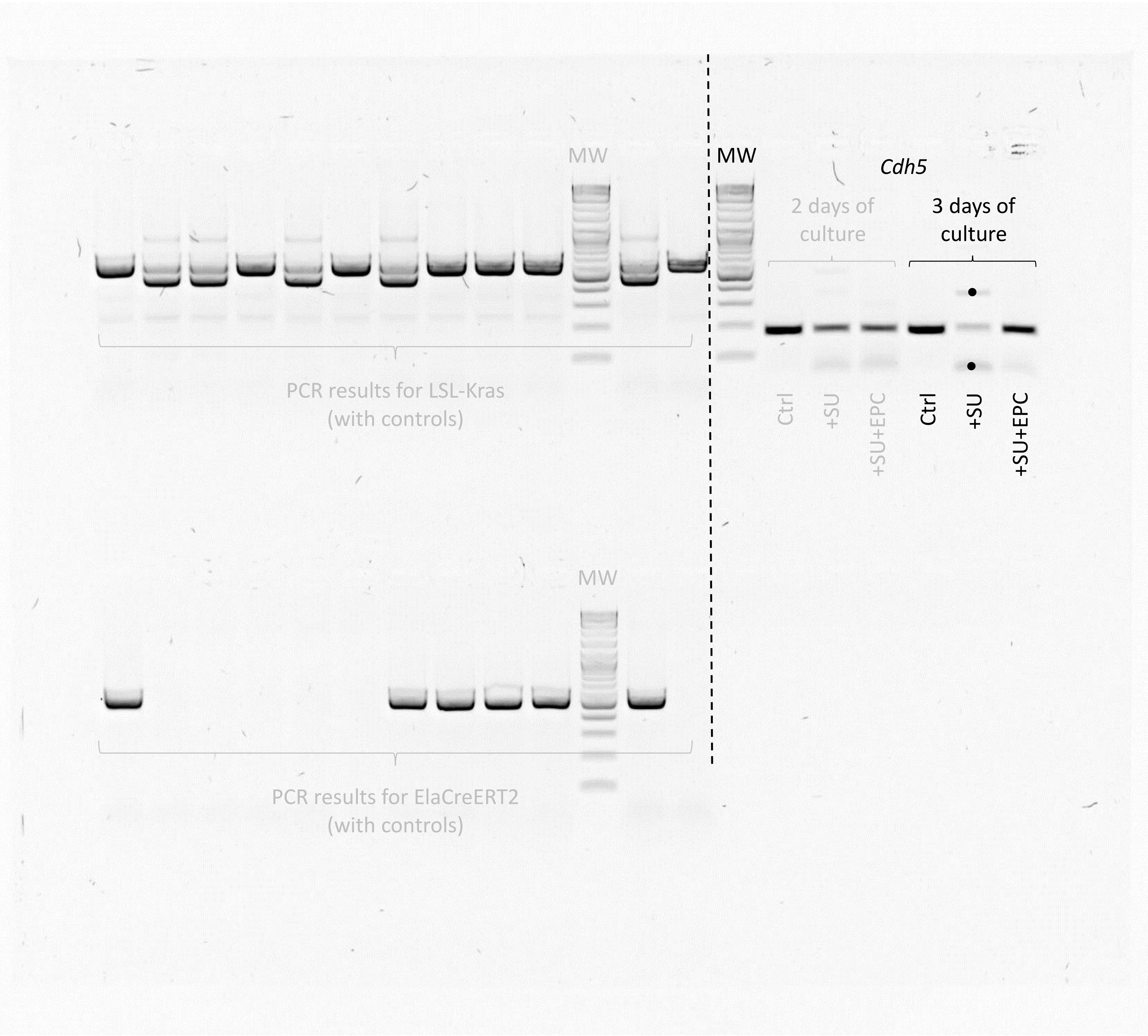

Figure S3: Full-length gel shown in Figure 2b.
The left side of the gel contains PCR samples of genotyping experiments (non relevant in this study). The PCR results of Cdh5 expression measured in explants cultured for 2 and 3 days in control medium (Ctrl), supplemented with SU5416 (+SU) and EPC (+SU+EPC), are found on the right side of the gel.

Table S1: Primers

Primer Name	Forward Sequence	Reverse Sequence	Primer efficiency (%)
Amy	5'-GTGGTCAATGGTCAGCCTTT-3'	5'-TTGCCATCGACCTTATCTCC-3'	98.8%
Car2	5'-TTGATGACTCTCAGGACAATGC-3'	5'-CTTGTGAGGCAGGTCCAATCTTC-3'	98%
Cdh5	5'-GGATGTGGTGCCAGTAAACC-3'	5'-ACCCCGTTGTCTGAGATGAG-3'	101.22%
Cpa	5'-CTCCTGACAAGGAGGAGCTG-3'	5'-ATAGTGCTCCCACTGGCTTG-3'	76.2%
E-Cadherin	5'-AGGGAGCTGTCTACCAAAGTG-3'	5'-GGAAACATGAGCAGCTCTGGG-3'	96.1%
Flk-1	5'-GCATGGAAGAGGATTCTGGA-3'	5'-CGGCTCTTTCGCTTACTGTT-3'	90.5%
Gcg	5'-GCACATTCACCAGCGACTACA-3'	5'-CGGTTCCTCTTGGTGTTCATC-3'	88%
Hnf1β	5'-GAAAGCAACGGGAGATCCTC-3'	5'-GACTGCCCAGGCCCCTGGTTCTGT-3'	92.2%
Ins2	5'-CAGGTGACCTTCAGACCTT-3'	5'-GGGTCTAGTTGCAGTAGTTC-3'	95%
Pecam	5'-ATAGGCATCAGCTGCCAGTC-3'	5'-TCCGCTCTGCACTGGTATTC-3'	91.6%
Prox1	5'-CCGACATCTCACCTTATTCAG-3'	5'-TGCGAGGTAATGCATCTGTTG-3'	104%
Ptf1a	5'-TGCCATCGAGGCACCCGTTC-3'	5'-TGAGCTGTTTTTCATCAGTCCAG-3'	89.6%
Rbpj	5'-GGTCCCAGACATTTCTGCAT-3'	5'-GGAGTTGGCTCTGAGAATCG-3'	91.1%
Rbpjl	5'-CAGAGCATGCCATCATCCTA-3'	5'-AGTCCCATGTAACCGCAGAC-3'	91.1%
Sox9	5'-CAAGACTCTGGGCAAGCTCTG-3'	5'-TCCGCTTGTCCGTTCTTCAC-3'	92.3%
β-Actin	5'-TCCTGAGCGCAAGTACTCTGT-3'	5'-CTGATCCACATCTGCTGGAAG-3'	100.3%

Table S2: Antibodies

Antibody	Supplier	Reference	Species	Dilution	Unmasking	Embedding
**Amylase**	Sigma	A8273	rabbit	1/300	+	gelatin
**E-Cadherin**	BD Biosciences	610182	mouse IgG2a	1/300	- or +	paraffin or gelatin
**Laminin-α1**	gift from T. Sasaki	/	rabbit	1/1,000	-	paraffin
**pan-Laminin**	Sigma	L9393	rabbit	1/200	- or +	gelatin
**PECAM**	BD Biosciences	550274	rat	1/100	-	gelatin
**PECAM**	Dianova	DIA310	rat	1/20	+	paraffin
**SPARC**	Santa Cruz	AON-1	mouse IgG3	1/300	-	gelatin
